# In Situ Observation of Micro-Patterned Elastomeric Surfaces: The Formation of the Area of Real Contact and the Influence on Its Friction and Deformation Behaviour

**DOI:** 10.3390/ma16196489

**Published:** 2023-09-29

**Authors:** Andreas Hausberger, Marina Pecora, Damien Favier, Elisabeth Rossegger, Martin Tockner, Thomas Ules, Matthias Haselmann, Sandra Schlögl, Christian Gauthier

**Affiliations:** 1 Polymer Competence Center Leoben GmbH, Sauraugasse 1, 8700 Leoben, Austria; elisabeth.rossegger@pccl.at (E.R.); martin.tockner@pccl.at (M.T.); thomas.ules@pccl.at (T.U.); ma.haselmann@gmail.com (M.H.); sandra.schloegl@pccl.at (S.S.); 2 Institut Charles Sadron, Université de Strasbourg, CNRS, F-67000 Strasbourg, France; mpecora@unistra.fr (M.P.); damien.favier@ics-cnrs.unistra.fr (D.F.); christian.gauthier@ics-cnrs.unistra.fr (C.G.)

**Keywords:** pre-sliding contacts, contact mechanics, in situ tribology, micro-patterned surfaces, thiol-acrylate-based photopolymers

## Abstract

Structured surfaces, which are the basis of the lotus blossom effect, have great potential to serve/operate as functionalised surfaces, i.e., surfaces with specific and/or adjustable properties. In the present study, the aim is to use micro-structured elastomeric surfaces to specifically influence the friction and deformation behaviours on the basis of the shape and arrangement of the structures. Thiol-acrylate-based photopolymers patterned via nanoimprint lithography were investigated by using an in situ tribological measurement set-up. A clear influence of the different structures on the surface’s friction behaviour could be shown, and, furthermore, this could be brought into relation with the real area of contact. This finding provides an important contribution to further development steps, namely, to give the structures switchable properties in order to enable the control of friction properties in a targeted manner.

## 1. Introduction

Taking inspiration from structures in nature has already produced a number of technological achievements, such as the lotus flower and the gecko foot effects [1,2,3,4,5]. Hierarchical structures have made it possible to reduce surface tension and to increase adhesion significantly. However, not only the type and shape of the structure, but also the structure material itself, play an important role on the surface property. Stimuli-responsive materials in combination with specific structures are of great interest to reversibly switch functions, such as surface energy, deformation resistance, and friction level [6,7,8,9,10]. These stimuli include temperature, light, humidity, pH value, solvents, electric or magnetic fields, and hardness and enable a controlled change of the surface structure [11,12,13,14]. These surface structures influence the real area of contact for two mating bodies and are, therefore, of great importance for tribological phenomena including friction and wear. This quantity is not only of interest to the scientific community, but, due to its impact on friction and wear, also for a wide variety of technical applications. Consequently, surface structuring as the means of adjusting the frictional characteristics has been widely studied in the past [15,16,17,18,19,20]. Kumar et al. transferred complex surface morphologies found in plant leaves onto viscoelastic substrates to gain insights for the design of sustainable (bio-inspired) and friction-tuneable technical surfaces [21]. High friction between the plunger and barrel also poses problems in medical syringes, where this may lead to problems in drug delivery. To solve this issue, Kasem et al. introduced surface textures on the plunger to reduce friction [22]. Low friction is also desired in most technical tribological systems to reduce energy costs and wear. Surface texturing is an effective way to achieve these goals without changing the material or lubricant. Zambrano et al. combined friction measurements of a surface textured rubber specimen with Reduced Order Modelling to compute optimal surface texture parameters that provide the highest friction reduction within a given parameter space [23]. One class of materials that comes into focus here are thiol-acrylate polymers, which exhibit excellent shape memory behaviour upon thermal triggering [24,25]. In order to be able to establish these shape-memory processes for selective control of frictional properties via surface textures, knowledge of the area of real contact, including the contact mechanics, is essential [17,26]. Here, the processes in the static friction area (pre-sliding) are of particular interest [27]. Excellent work has already been carried out in the field of single asperities, showing a decrease in the area of real contact with an increase in shear stress [28]. For this purpose, corresponding models were developed by Mindlin and Cattaneo [29,30]. In addition, there are well-developed contact models by Persson and Popov in relation to rubber friction available [31,32]. A large part of these studies has been carried out on polydimethylsiloxane (PDMS) [33,34]. In situ tribological investigations are always coupled to mathematical descriptions in order to relate the local processes in the contact to the resulting forces and stresses [35,36,37]. Many of these studies also focus on hemispherical single-point contacts, as the contact conditions are easier to control. With structured surfaces, there is still a clear need for further research due to the more complex contact conditions, especially through the combination of the material and type of structure [38].

In this paper, micro-structured elastomeric surfaces are investigated to influence the friction and deformation behaviours on the basis of the shape and arrangement of the surface structures. The novelty of this work lies in the materials used and the application of shape memory processes to selectively adjust the friction properties by controlling the actual area of contact.

## 2. Materials and Methods

### 2.1. Materials and Chemicals

**Figure 1:**
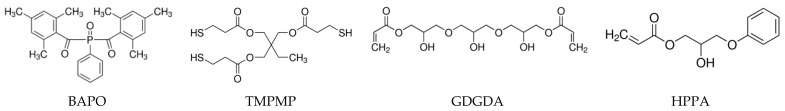
Chemical structures of the photoinitiator, the acrylate monomers, and the thiol.

The photoinitiator BAPO (phenylbis(2,4,6-trimethylbenzoyl)phosphine oxide) and the two selected acrylates 2-hydroxy-2-phenoxypropyl acrylate (HPPA) and glycerol 1,3-diglycerolate diacrylate (GDGDA) were supplied by Sigma Aldrich (USA). The stabiliser (Miramer A99) was obtained from Miwon Specialty Chemical (Korea), and the trimethylolpropane tri(3-mercaptopropionate (TMPMP) was kindly provided by Bruno Bock Chemische Fabrik (Germany). All chemicals were used as received. The chemical structures of the main components are depicted in Figure 1.

### 2.2. Preparation of the Resin

A total of 2 mol% phenylbis(2,4,6-trimethylbenzoyl)phosphine oxide was dissolved in HPPA (65 mol%), GDGDA (23 mol%) and 0.5 wt% Miramer A99 under stirring at 50 °C. After cooling down to room temperature, 10 mol% TMPMP was added and stirred for an additional 10 min.

### 2.3. Surface Production and Analytics

**Figure 2:**
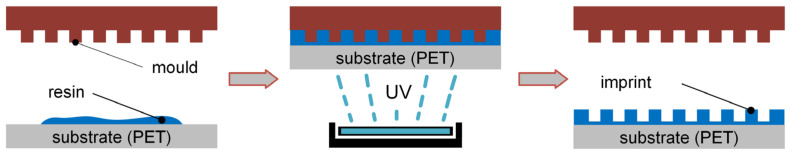
Production process of thiol-acylate structures based on nanoimprint lithography.

The structures were produced by nanoimprint lithography, which is schematically depicted in Figure 2. The resin (200 µL) was placed on a polyethylene terephthalate (PET) substrate (thickness of 10 µm), and subsequently, the mould was pressed onto it. Via UV-light irradiation (through the transparent PET substrate, Omnicure S2000, 70% intensity, 1 min duration), the resin solidifies, and the mould can be removed.

**Figure 3:**
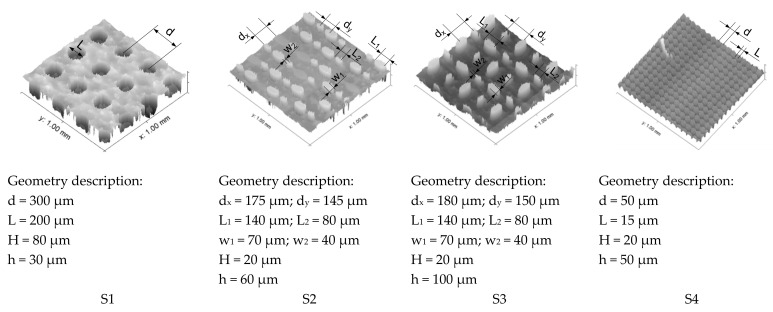
Topography information as obtained from confocal microscopy of the investigated surfaces named S1–S4 with the correspondent geometrical information.

In total, four differently structured surfaces were produced for the investigations. For the determination of the topographical information of the structures, a confocal microscope Microprof MPR1080 (Fries Research and Technology (FRT) GmbH., Bergisch Gladbach, Germany) was used, and in Figure 3, the topographical images are visualised.

For the description of the structures, the distance *d* between the structure elements in x and y direction, the elements length *L*, the element width *w*, and the element thickness *H*, as well as the base layer thickness h, were defined as shown in Figure 3. For the structures S2 and S3, due to two different elliptic element sizes, the indexes 1 and 2 are used. The different surface structures that were prepared are (i) structure S1, which represents a surface with holes and, in principle, has nominally larger contact zones and also the highest structure thickness of *H* = 80 µm; (ii & iii) the structures S2 and S3, which represent a raised structure and differ from each other in the orientation of the elliptical elements within the series; and (iv) the structure S4, which represents a lattice-structured surface.

The surface free energy of the imprints was determined by static contact angle measurements on a drop shape analysis system DSA 100 (Krüss, Hamburg, Germany). Two µL droplets of deionised water and diiodomethane were used as test liquids. All contact angles were obtained by calculating the arithmetic average from ten different points of each sample, and the surface free energy was then calculated according to Owens, Wendt, Rabel and Kaelble [39,40,41] and is reported in Table 1.

**Figure 4:**
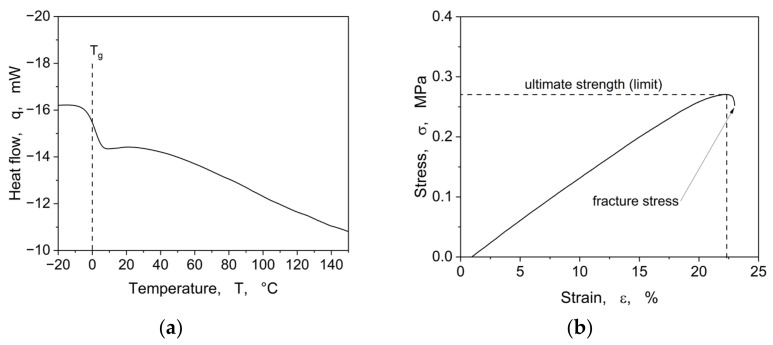
Basic thermal and mechanical characteristics of the non-structured resin, showing in (**a**) the thermal behaviour and in (**b**) the mechanical behaviour.

Differential scanning calorimetry (DSC) measurements were carried out on a Perkin Elmer DSC 8000 under a nitrogen atmosphere. A temperature program ranging from −20 to 150 °C, with a heating rate of 20 °C/min, was applied. The glass transition temperature (*Tg*) was calculated from the second heating cycle with Pyris software, version 13.3.1, and the measurement representative is shown in Figure 4a.

Tensile tests were conducted on a ZwickRoell (Ulm, Germany) Z1.0 static materials testing machine with a crosshead speed of 250 mm min^−1^. In Figure 4b, one stress–strain curve is depicted.

The above shown DSC measurement (see Figure 4a) displays a glass transition temperature at 0 °C. From the stress–strain curve (see Figure 4b), an ultimate strength *σ_u_* of 0.27 MPa at 22% elongation and an elastic modulus of 1.5 MPa for the non-structured resins could be determined.

### 2.4. In Situ Tribometer

**Figure 5:**
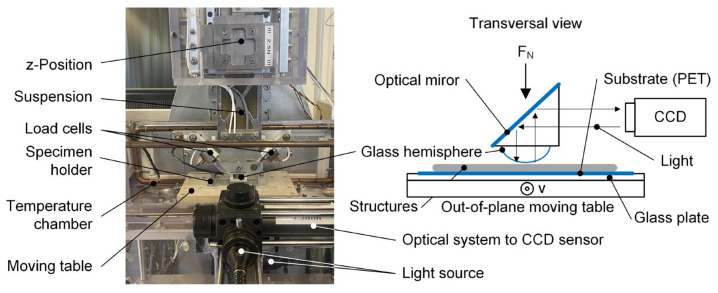
Visualisation of the in situ tribometer with optical path and sketch of the contact set-up.

The in situ tribometer (shown in Figure 5) employed in these investigations is an in-house development and has already been described several times in detail by Gauthier et al. [42,43]. For this present optical path, the measurement principle consists of using a glass hemisphere with a diameter of 25.8 mm (Borosilicate BK7 precision lenses, Newport^®^, Irvine, CA, USA), generating the contact with the structures, and, by means of a mirror placed above the glass hemisphere, the contact formation can be inspected in situ by a CCD sensor at the end of the optical path. The load cell has a force range of 0.01–2.5 N. The possible velocity range is 0.001–1 mm/s.

### 2.5. Linear and Cyclic Motion Testing

**Figure 6:**
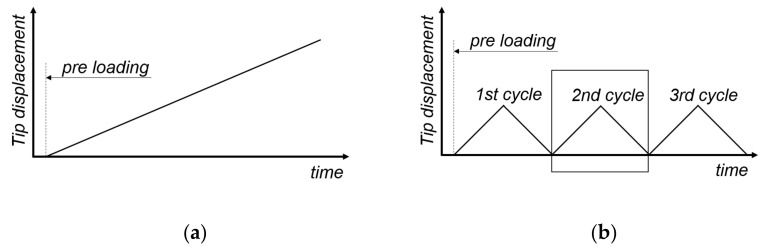
Schematic test sequence of the tip displacement over time for (**a**) linear movement and (**b**) cyclic movement with representation of the three cycles.

Due to the fact that these structures have never been tribologically investigated elsewhere, two different measurement procedures were implemented for the present study. The first consisted of a linear movement to investigate the development of the tangential force *Q* in the pre-sliding region and the transition into the dynamic sliding regime. This would reveal information about the structure deformation and behaviour in the steady state. Here, four different loads (0.05, 0.1, 0.2, and 0.5 N) and two velocities (0.001 and 0.01 mm/s) were chosen. The displacement was set between 0.2 and 0.5 mm, and the schematic test sequence is shown in Figure 6a. The second procedure consisted of cyclic movements to investigate the hysteretic components of the pre-sliding and to obtain information about the dissipated energy and specific damping capacity in contact. A custom script was specifically developed for the tribometer in the context of this measurement program, enabling the recording of three hysteresis loops, along with their corresponding parameter sets (described in Figure 6b). The investigations were carried out at one amplitude (0.1 mm), chosen based on the evaluated pre-sliding regime of the linear movement experiments, four loads (0.05, 0.1, 0.2, and 0.5 N), and one velocity (0.01 mm/s). A short constant preloading time (<2 s) was used to keep structural and material-based changes for all experiments the same. In both measurement methods, the tangential force *Q* was directly measured via the load cell, and the coefficient of friction *COF* was calculated by forming the ratio between the tangential force and the applied normal load. All measurements (linear and cyclic) were performed under ambient conditions (25 °C and 48% RH).

### 2.6. Area of Real Contact Calculations

The concept used in this paper to determine the area of real contact *A_R_* is based on employing the generated contrast between the glass hemisphere and the polymeric surface via the optical setup, as described in Figure 5 and by Gauthier et al. [42,43]. When structures become in contact with the glass hemisphere, the contrast decreases and allows more light penetration into the CCD sensor. These changes in brightness were used to distinguish contact from non-contact zones. Due to different structures and associated light conditions, a separate contrast calibration was carried out for each structure, using two light sources in combination with the camera setting (brightness and contrast).

**Figure 7:**
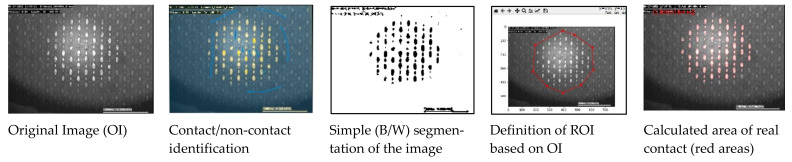
Image processing routine to calculate the area of real contact using ilastik^®^ and Python script, using structure S2 as an example.

As a basis for processing the in situ images, AVI film data were synchronised with the position of the linear motor within the VirtualDub software, version 1.10.4 (Free Software Foundation Inc., Boston, MA, USA). This allowed for assigning the friction input prevailing in each frame to the image information and preparing it for image analysis. For this purpose, the ilastik^®^ software, version 1.3.3, was used for supervised pixel-level classification, and, with the help of assisted machine learning, a pixel assignment of the structures and thus the real area of contact determination was carried out [44]. The assigned contact areas were converted to black and white for further processing, and the contact area was calculated in a Python script by selecting the region of interest (ROI) on a pixel basis. The image processing procedure is represented in Figure 7.

### 2.7. Evaluation of the Contact Parameters

Besides the calculation of the area of real contact between the structures and the glass hemisphere, other parameters, such as the tangential stiffness (for both linear and cyclic tests) and the dissipated energy (for cyclic tests only), are used to gain more information about the deformation and damping behaviour of the structures. In this context, a specific damping capacity ψ can be calculated, which allows a statement regarding the mechanical dissipation during a dynamic loading. The dissipated energy ∆*W* is calculated, using numerical integration with a Python script, as the hysteresis loop area of the second of the three recorded cycles. The dissipated energy is normalised with respect to the maximum stored energy per load cycle, and the corresponding damping capacity is calculated according to [45,46]. Equation (1) represents the tangential stiffness *K_t_*, using the measured maximum tangential force *Q_m_* and maximum displacement *δ_m_*.

(1) $$K_{t}=\frac{Q_{m}}{\delta_{m}}$$

The specific damping capacity is calculated (Equation (2)) using the dissipated energy ∆*W* and the maximum tangential force *Q_m_*.

(2) $$\psi =\frac{\Delta W}{1/2Q_{m}\delta_{m}}=\frac{2\Delta W\;K_{t}}{Q_{m}^{2}}$$

## 3. Results

### 3.1. Linear Motion

**Figure 8:**
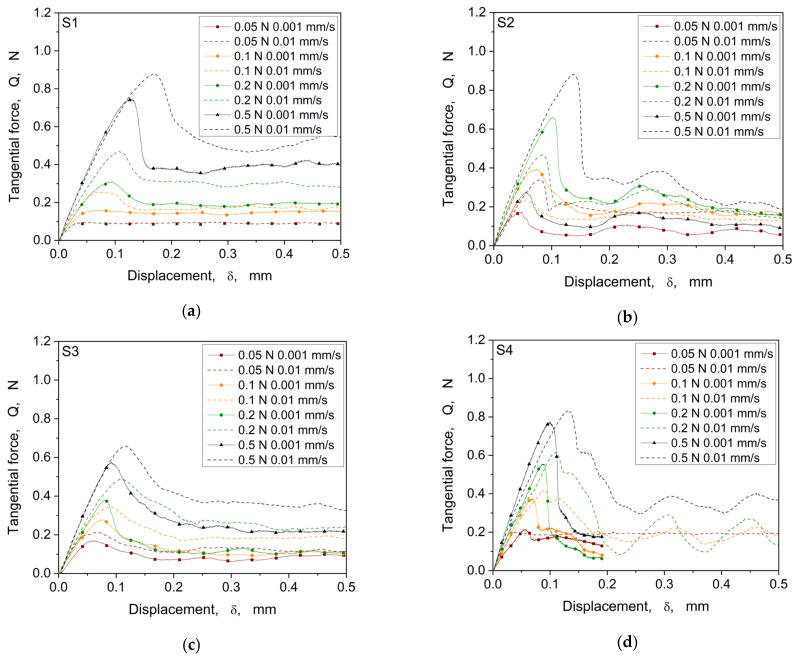
Comparison of the tangential force vs. tangential displacement curves at different velocities and normal load levels of the four structures S1 (**a**), S2 (**b**), S3 (**c**), and S4 (**d**).

The evolution of the tangential force as a function of the tangential displacement of the four structures, for a test velocity of 0.001 (continuous line) and 0.01 mm/s (dashed line) and for different values of normal load, is represented in Figure 8.

**Figure 9:**
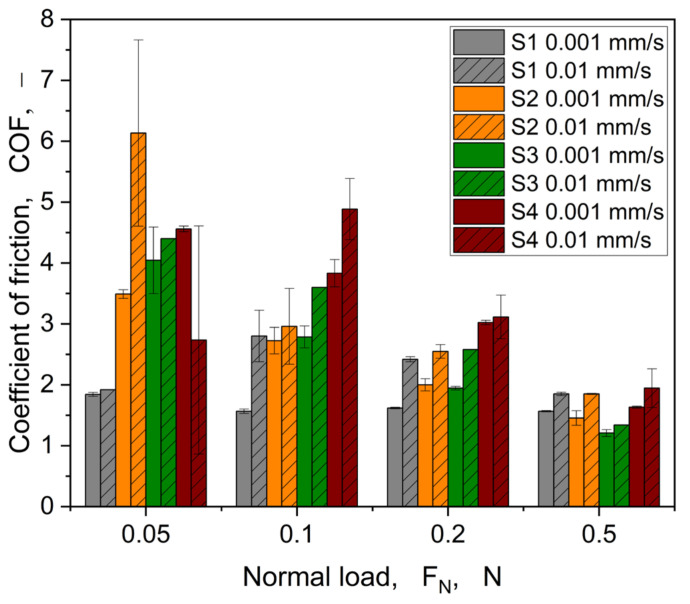
Representation of the coefficient of friction as a function of velocity, normal load F_N_, and structure.

In general, as observed in the curves shown in Figure 8, we can distinguish the pre-sliding regime, in which the tangential force increases monotonically with displacement and a dynamic sliding regime, in which sliding begins, where the tangential force drops until steady state. Thus, the tangential load required to initiate the motion (in the pre-sliding regime) is higher than the one needed to keep the movement, which is consistent with the very early observations on the distinction between static and dynamic friction and which explains why the static friction coefficient is usually higher than the dynamic one [47]. In some cases (Figure 8b,c), fluctuations are observed in the dynamic regime, which can be ascribed to structure damage that locally modifies the morphology of the surface and, thus, the contact conditions. For the follow-up analysis, the focus lies on the pre-sliding regime within a clear load, and the velocity dependency is visible for all samples, especially for the higher loads, 0.2 and 0.5 N, which show a strong increase of the tangential force until the maximum static friction is reached. In Figure 9, the coefficient of friction calculated at the maximum tangential force is represented as a function of the normal load.

**Figure 10:**
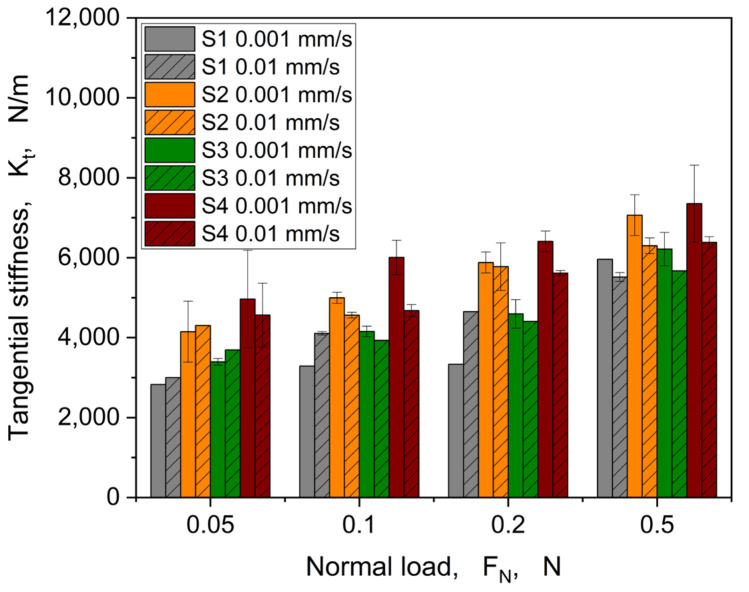
Tangential stiffness as a function of the normal load for all samples and both velocities (0.001 and 0.01 mm/s).

The coefficients of friction globally decrease with increasing normal load for all samples. Furthermore, the coefficient of friction increases with increasing speed, when considering a normal load of more than 0.1 N. At the minimum normal load of 0.05 N, the speed dependency is not clearly visible due to difficulties with contact formation between structure and glass hemisphere because of reaching a lower load sensor limit. This is also reflected by the high standard deviation of S2 and S4 at a velocity of 0.01 mm/s. However, the characteristics vary from structure to structure. Only structure S1 shows an unpronounced load dependency for the lower velocity of 0.001mm/s. This could be attributed to the different geometrical composition of the structure S1, which has holes instead of pillars, like the structures S2–S4. In addition to the examination of the maximum values in the static friction regime, an evaluation of the tangential stiffness is helpful with regard to an assessment of the structures. For this purpose, Equation (1) is used to calculate *K_t_* for all structures, and this parameter is shown in Figure 10 as a function of the normal load.

Overall, it can be seen that for all structures, the tangential stiffness increases with the normal load. When considering the influence of velocity, it becomes apparent that there is no clear trend visible by involving the error bars. Considering a viscoelastic material behaviour, the bulk stiffness should be increasing with higher velocities. In our case, this is not generally observable. Furthermore, a slight decrease in tangential stiffness with increasing velocity in the majority of structures is visible (cp. Figure 10). Some deviations are visible at a minimum load of 0.05 N and in general for S1, as already described with regard to the evolution of the coefficient of friction (cp. Figure 9). Already, a slight difference between the structures could be revealed. Especially the mean values of S2 and S4 show the highest tangential stiffness concerning load and velocity. This could be explained by the overall thickness of structures in combination with the base layer thickness (approx. 60–70 µm) in comparison to the structures S1 and S3, showing an overall thickness (*H + h*) of approx. 110–120 µm. Therefore, S1 and S3 are also on a comparable tangential stiffness level.

**Figure 11:**
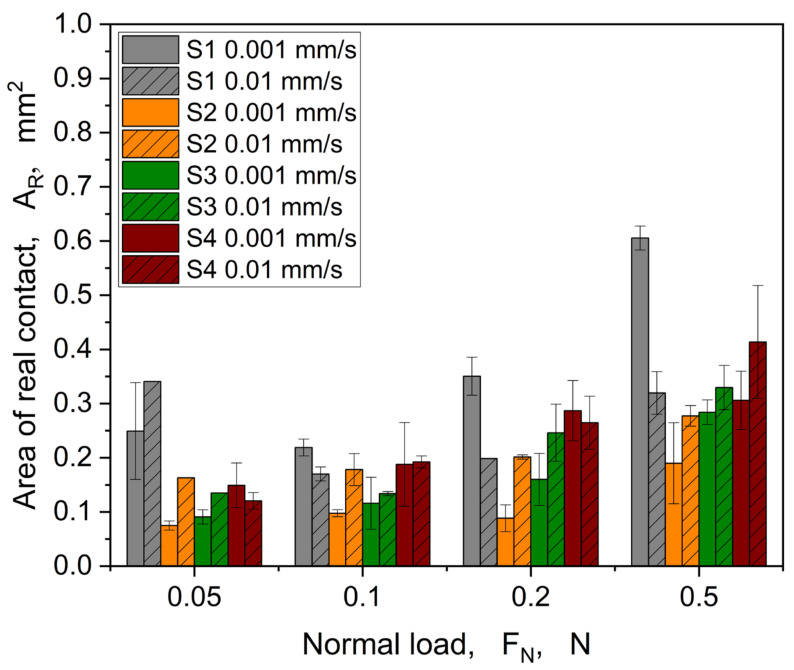
Comparison of the area of real contact calculated at the maximum tangential force level in relation to the different structures.

**Figure 12:**
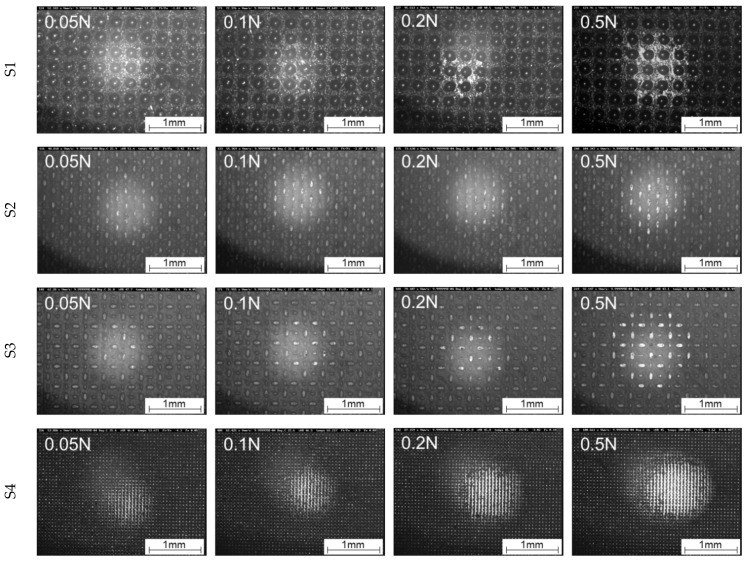
Comparison of the contact formation of S1, S2, S3, and S4 as a function of the load under linear motion at maximum static friction force at a velocity of 0.001 mm/s.

**Figure 13:**
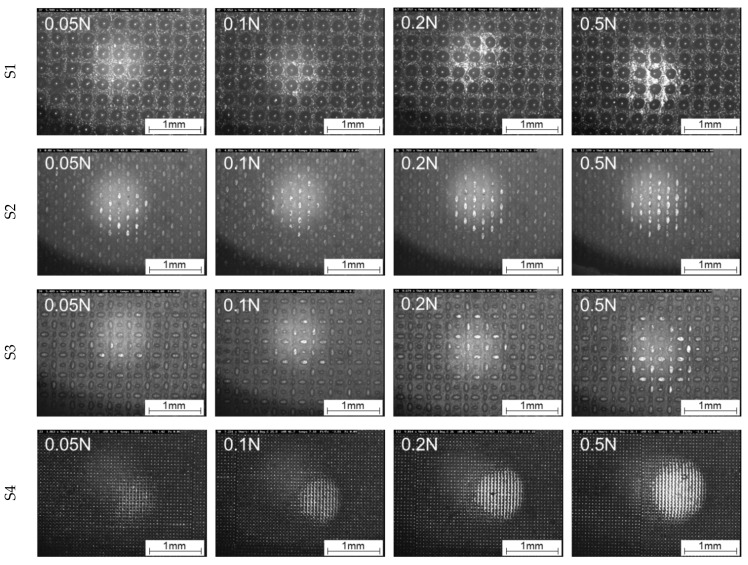
Comparison of the contact formation of S1, S2, S3, and S4 as a function of the load under linear motion at maximum static friction force at a velocity of 0.01 mm/s.

The calculated area of real contact shows generally an increase with increasing load (see Figure 11), which is consistent with the theory of the contact between a rigid body and an elastic solid proposed by the Hertz, for which the contact radius cubed is proportional to the applied normal load [48]. As expected, the different structures show clear deviations from each other in terms of level and progression, which is also depicted in Figure 12 (0.001 mm/s) and Figure 13 (0.01 mm/s), showing in situ images at the maximum tangential force.

In the in situ images from Figure 12 (0.001 mm/s) and Figure 13 (0.01 mm/s), it is also clearly recognisable at which normal load first damage to the structures occurs due to the shear deformation. In general, the first changes in the shape of the structures can be seen from a normal load of 0.2 N at a velocity of 0.001 mm/s and at 0.1 N for the velocity of 0.01 mm/s. To illustrate a better impression of the evolution of the area of real contact in correlation with the tangential force and damage evolution, in situ videos at 0.1 N are provided in the Supplementary Materials Section, for 0.001 mm/s (Videos S1a, S2a, S3a, and S4a) and 0.01 mm/s (Videos S1b, S2b, S3b and S4b).

### 3.2. Cyclic Motion

**Figure 14:**
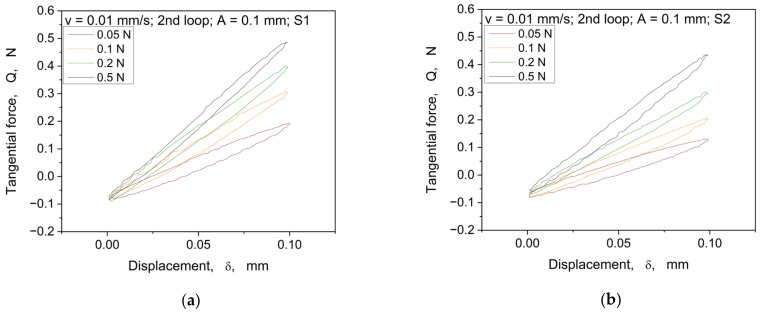

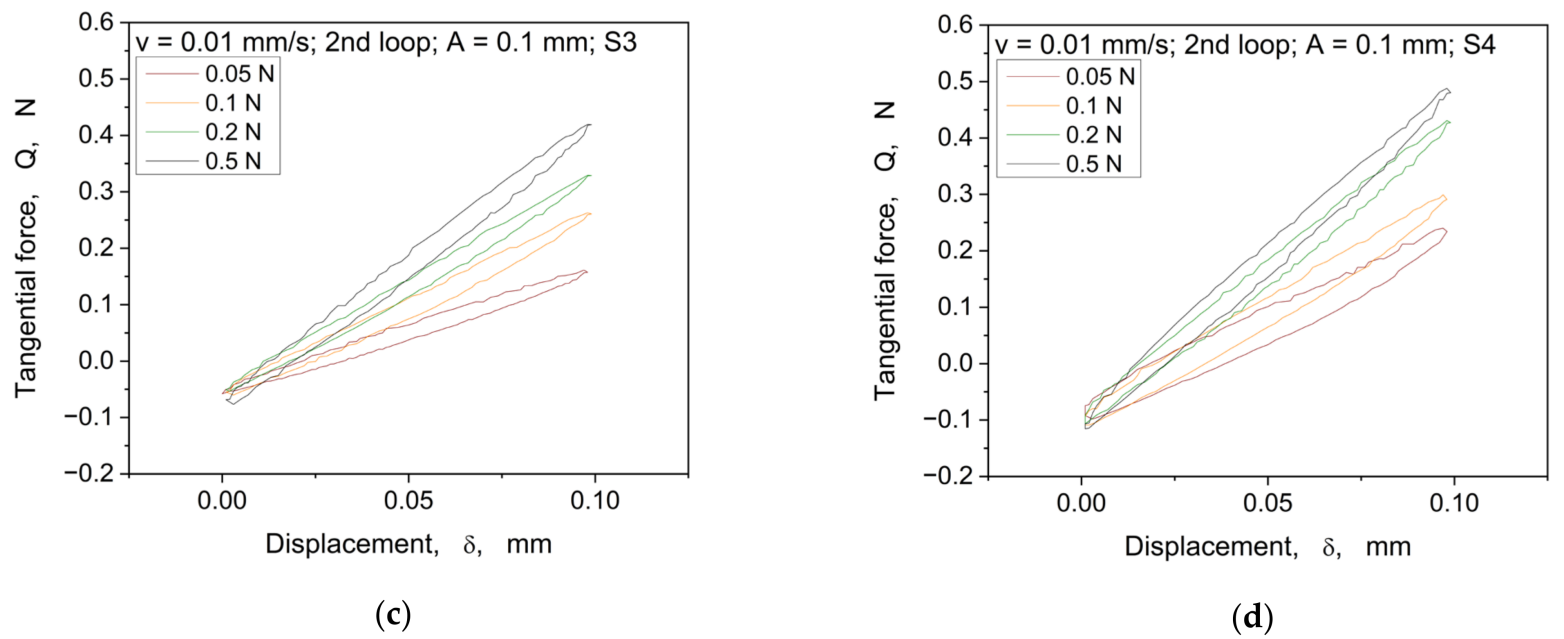
Comparison of the 2nd hysteresis loop showing the evolution of the tangential force over displacement at an amplitude of 0.1 mm and a velocity of 0.01 mm/s for the structures S1 (**a**), S2 (**b**), S3 (**c**), and S4 (**d**).

The pre-sliding hysteresis for four structures is shown in Figure 14. Due to the better representability and excellent reproducibility of the hysteresis, only the second loop from the individual measurements is shown below.

The hysteresis curves show similar changes of the slopes in relation to the normal load dependence, as in the monotonic tests (cp. Figure 8). With an increase in the normal load, the hysteresis loop rises. Furthermore, with the increase in the normal load, it can be seen that the hysteresis curves change their shapes (e.g., roundness), which also seems to be dependent on the different structures.

**Figure 15:**
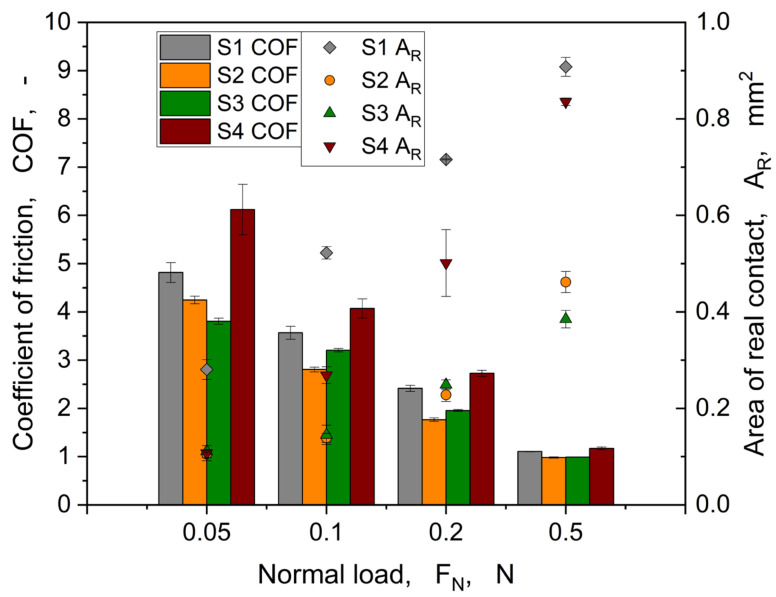
Evolution of the coefficient of friction and the area of real contact as a function of the normal load for the structures S1, S2, S3, and S4.

Figure 15 shows the coefficient of friction and the real area of contact, evaluated at the maximum tangential force *Q_m_*, against the normal load for the samples S1, S2, S3, and S4.

The trend shown in Figure 15 is quite comparable to the coefficient of friction behaviour for linear motion experiments (cp. Figure 9), in which a decrease of *COF* was observed as the normal load increased. At a normal load of 0.5 N, the coefficient of friction is roughly the same for all samples, reaching a value of around 1. Regarding the area of real contact, a steady increase was observed for all structures with the normal load. At the normal load of 0.05 N, only S1 shows a higher *A_R_*, which could be explained with the hole-patterned structure showing, in principle, a higher contact area. This behaviour is also reflected in the linear motion experiments (see Figure 11). Furthermore, two trends are visible: (i) structures S1 and S4 show higher *A_R_* compared to the other structures (S2 and S3), and (ii) the difference in height for S1 and S4 reduces with increase in the normal load.

**Figure 16:**
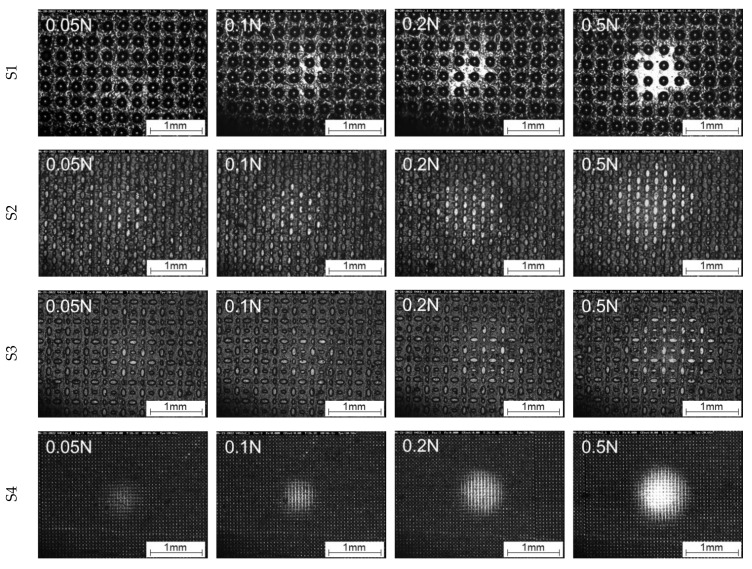
Comparison of the contact formation of S1, S2, S3, and S4 as a function of the load under cyclic motion at maximum static friction force.

From Figure 16, S1 shows a larger area of real contact compared to S2, S3, and S4. For the load ranges of 0.05 to 0.2 N, a higher area of real contact also correlates with a higher maximum tangential force. S4 shows, especially at a normal load of 0.5 N, a quite comparable level of area of real contact and maximum tangential force with S1. It seems that for both structures, the maximum load-bearing capacity is reached due to alignment independently from structure type. This hypothesis can also be supported by the different structure and base layer thicknesses. For example, although S1 has a low base layer thickness (30 µm), it has the maximum structure height (80 µm) of all structures. For S4, the structure thickness is only 20 µm, which is transferred to the base layer with a thickness of 50 µm at 0.5 N. Moreover, Figure 16 (S4, 0.5 N) shows an almost disappearance of the structures due to high compression. A closer look at samples S2 and S3, which differ only in the orientation of the elliptical structures with respect to the sliding direction, shows that this has only a minor effect on the area of real contact and the coefficient of friction. Both structures have the same structural thickness (20 µm), but sample S3 has a base layer thickness that is almost twice the size for S2. Here, only within the linear motion experiments, the tangential stiffness seems to be slightly higher for S2 (*h* = 60 µm) than for S3 (*h* = 100 µm) (cp. Figure 10). This could be attributed to the increasing influence of the more rigid PET substrate.

**Figure 17:**
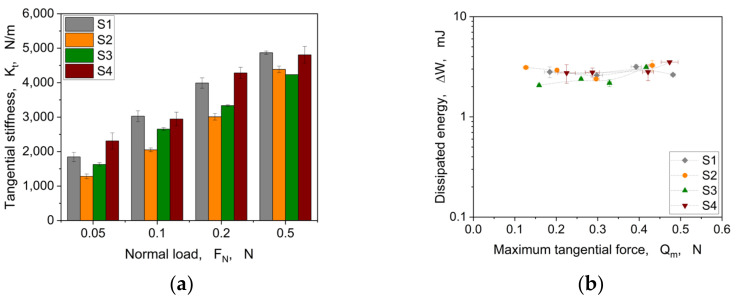
Tangential stiffness under different normal loads (**a**) and evolution of the dissipated energy ∆W as a function of the maximum tangential force Qm under cyclic loading (**b**).

Regarding the contact parameters for the cyclic tests, the evaluated tangential stiffness based on Equation (1) is shown in Figure 17a, and the calculated dissipated energy from the hysteresis area with the corresponding maximum tangential force is depicted in Figure 17b.

**Figure 18:**
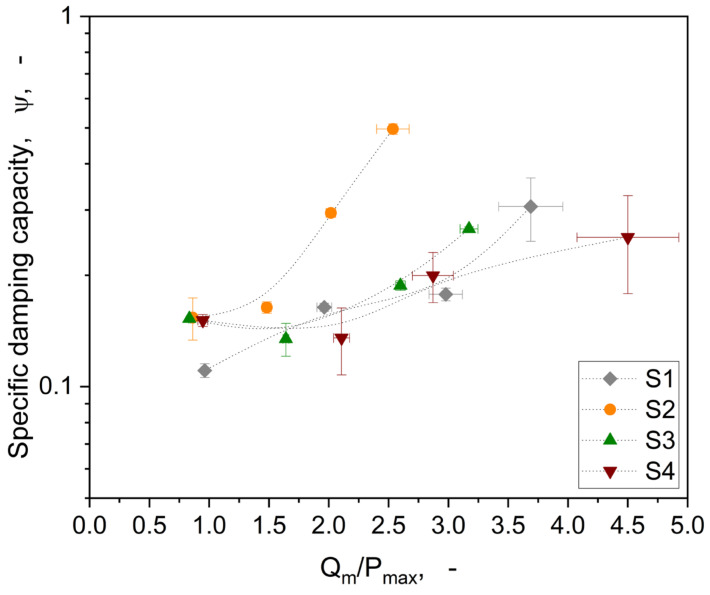
Representation of the specific damping capacity ψ in relation to the normalised maximum tangential force by the normal load. The lines represent only the data trend.

Visualising the tangential stiffness over the normal load, an increase of *K_t_* with higher loading is shown (see Figure 17a). The same trend is also apparent for the linear motion in relation to *K_t_* visible (cp. Figure 8). Also, here, two groups are visible: (i) S1 and S4 show a higher overall *K_t_* compared to (ii) S2 and S3. The dissipated energies (cp. Figure 17b) of the hysteresis loops do not show a pronounced load dependency nor significant differences between the structures. This means that there is no change in contact conditions within the load range used due to the respective structures. However, the structures show different maximum tangential forces based on the represented normal load (cp. Figure 14). Figure 18 shows the specific damping capacity in relation to the tangential force normalised to the applied normal load.

Considering the dynamic damping behaviour of the structures using the specific damping capacity, Figure 18 shows indeed quite reasonable trends and differences among the investigated structures. Each structure follows its own normal load dependency with regards to the damping behaviour. Especially for S2, a quite interesting trend is exposed. This structure showed the highest specific damping capacity, at least for 0.2 and 0.5 N applied normal loads, considering a rather low coefficient of friction (cp. Figure 15). A quite similar behaviour was also observed within the linear pre-sliding investigations revealed by the tangential stiffness (cp. Figure 10). This kind of ellipsoidal structure with orientation normal to the deformation direction represents the most promising characteristics, considering the incorporation of stimuli-responsive properties. S3 also shows a slight trend in this direction, but is underrepresented compared to S2. As already mentioned regarding the evolution of the area of real contact (cp. Figure 15), the structures S1 and S4 show an alignment to their *ψ* values with higher normal load. This implies that the load bearing capacity is reached, showing a bulk-related damping behaviour rather than a surface structure-related one.

## 4. Conclusions

In the present work, different micro-structures based on thiol-acrylates were successfully produced by means of nanoimprint lithography and investigated with regard to the pre-sliding behaviour. The studied structures showed different responses, which could be correlated by means of the in situ observations. Furthermore, due to the small sizes and the high adhesion, the structures showed a certain wear and tear at certain loads, which led to the destruction of the structures. By observing the load limits in relation to the shear deformation, clearly demonstrable differences of the structures and the influence on the static friction behaviour could be shown. The shape of the structure compared to the area of real contact dominates the magnitude of the friction force and, thus, the level of damping capacity. Furthermore, the load limit at 0.5 N also indicated the same behaviour, as the friction forces and dissipated energies settle at comparable levels. Nevertheless, considering the aforementioned results, S2 was identified as a promising candidate for further investigations concerning switchable surface properties. This structure shows a unique deformation and damping behaviour based on shape and orientation, which would be needed to be introduced in the upcoming work related to stimuli-responsive properties. It is believed that by controlling the damping and deformation behaviour of such structures, a selective frictional response would be generated, introducing new properties that could be easily transferred as a layer or coating via imprint lithography for, e.g., soft robotic applications with controlled haptic properties in the health care sector.

## Figures and Tables

**Table 1:** Comparison of the surface energies measured on the different structures.

Sample	Disperse Part [mN/m]	Polar Part [mN/m]	Total Surface Energy [mN/m]
S1	35.77 ± 1.10	3.80 ± 0.28	39.57 ± 1.38
S2	33.55 ± 1.25	2.80 ± 0.25	36.35 ± 1.50
S3	34.61 ± 0.94	5.14 ± 0.24	39.75 ± 1.18
S4	30.30 ± 1.32	3.50 ± 0.30	33.80 ± 1.63

## Data Availability

Not applicable.

## Supplementary Materials

The following supporting information can be downloaded at: https://www.mdpi.com/article/10.3390/ma16196489/s1, Video S1a: S1_0.1N_0.001 mm/s; Video S2a: S2_0.1N_0.001 mm/s; Video S3a: S3_0.1N_0.001 mm/s; Video S4a: S4_0.1N_0.001 mm/s; Video S1b: S1_0.1N_0.01 mm/s; Video S2b: S2_0.1N_0.01 mm/s; Video S3b: S3_0.1N_0.01 mm/s; Video S4b: S4_0.1N_0.01 mm/s.
